# Editorial: Medical big data in cancer research

**DOI:** 10.3389/fmolb.2024.1395607

**Published:** 2024-03-13

**Authors:** Jingxin Mao, Ke Zheng, Xiong Weng

**Affiliations:** ^1^ Chongqing Medical and Pharmaceutical College, Chongqing, China; ^2^ College of Pharmaceutical Sciences, Southwest University, Chongqing, China; ^3^ Department of Endocrine and Breast Surgery, The First Affiliated Hospital of Chongqing Medical University, Chongqing, China; ^4^ Centre for Cardiovascular Science, Queen’s Medical Research Institute, University of Edinburgh, Edinburgh, United Kingdom

**Keywords:** big data, biomedical research, healthcare, cancer, database

According to the data of the Global Cancer Report, the incidence rate and mortality rate of cancer was reached 19.29 million and 9.96 million respectively in 2020. Cancer has become the primary problem affecting the life and health of all human beings, posing a serious threat to human life and health (Xi and Xu, 2021). With the increase in the number of cancer patients, the clinical medical data generated is also becoming increasingly large. As a traditional medical record model, doctor workstations can no longer meet the needs of researchers in mining, analyzing, and utilizing clinical data (Bray et al., 2018). In the field of cancer research, big data analysis plays a crucial role. Its profound heritage lies in its ability to provide rich case data, enabling medical researchers to understand the hidden connections and patterns between cases (Hassan et al., 2022). Moreover, big data analysis can provide strong support for medical researchers to explore potential new drugs and treatment methods. Through deep mining and analysis of the vast disease database, we can discover biomarkers, gene variations, and drug responses closely related to diseases, providing a solid foundation for the develop varies of new drugs and the innovation of treatment methods (Mao et al., 2023).

**FIGURE 1:**
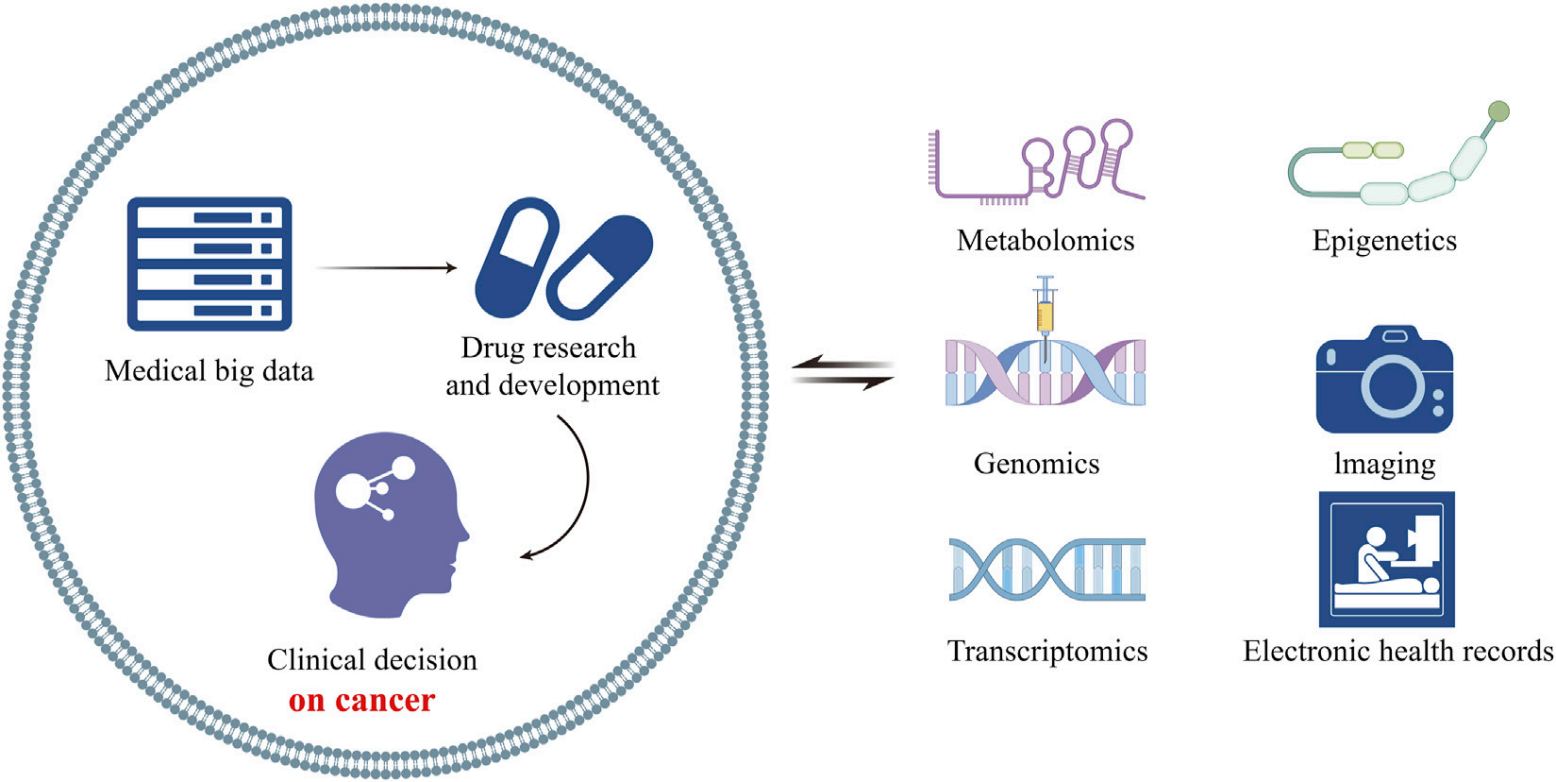
The relationship of medical big data and cancer research (By Figdraw).

In addition, big data analysis has demonstrated excellent capabilities in drug safety and efficacy evaluation (Krallinger et al., 2010). By deeply analyzing a large amount of clinical trial data and real-world treatment data, we can accurately evaluate the side effects, efficacy, and differences in treatment efficacy of drugs in different populations, providing valuable guidance for rational drug use and personalized treatment (Tabib et al., 2020). Taken together, the development of medical big data enables molecular biological analysis at the micro level, such as metabolomics, proteomics, epigenetics and other technologies (Figure 1, by Figdraw, ID:TUSIYd5de0). Combined with the molecular mechanism of disease, the relationship between gene protein (enzymes, receptors, channels, signaling molecules, etc.) and cancer can be summarized from multi sample and big data analysis, providing a new scientific basis for the creation of new drugs and therapies. Therefore, totally 5 original research manuscripts by universally acknowledged authors which was collected on the related fields.

Borisov et al. proposed a novel gene expression data normalization method called Shambhala, which can present normalized data in a unified format, transforming each expression profile into a predefined universal format. It was found that the generalization phenomenon of human ribonucleic acid (RNA) profiles, which showed a significant decrease in platform specific clustering while strongly preserving the overall tissue-specific clustering features.

Eng et al. investigated the association between oxidative stress, deoxyribonucleic acid (DNA) damage, and genetic changes in benign thyroid lesion in one lobe (BTG) to papillary thyroid cancer (PTC) transformation. Due to the increase in reactive oxygen species (ROS) levels, the oxidative DNA damage intensifies, which may indicate the transformation of BTG to PTC. This may be achieved through mutations in genes related to the mitogen-activated protein kinase (MAPK) signaling pathway and stress activated MAPK/c-Jun N-terminal kinases (JNK) cascade reactions.

Kang et al. established a model based on immune related gene expression using the expression of immune related genes and calculated the scores for each sample. Analyzed the correlation between the model and clinical information, immune infiltration, drug response, and biological pathways. It was revealed that cancer related pathways, including immune related pathways, are significantly activated in high scoring samples, and some drugs have significantly lower IC_50_ values than low scoring drugs. The model developed based on immune related genes is robust and reflects various states of colorectal carcinoma (CRC), which may be a potential clinical indicator.

Zhou et al. used machine learning models to screen metabolomic features from cross queue datasets of colorectal cancer (CRC) and colorectal adenoma (CRA). Then select a CRC and CRA dataset from the CuratedMetagenomicData database to meet the requirement of having both metabolomic and clinical data. Integrating cross queue common identification of microbial characteristics with clinical features helps to construct a stable diagnostic model for early non-invasive screening of CRC and colorectal adenoma CRA.

Shi et al. investigated the shared genes between rheumatoid arthritis (RA) and lung adenocarcinoma (LUAD) through differential analysis and weighted gene co expression network analysis (WGCNA). Subsequently, COX regression and least absolute shrinkage and selection operator (LASSO) analysis were used to screen for genes significantly associated with survival. Verify the expression levels of candidate genes using qRT-PCR and Western blot techniques. The research results indicate that RA and LUAD share common physiological and pathological processes and molecular characteristics. Rheumatoid arthritis and lung adenocarcinoma (RALUADS) is expected to become an excellent prognostic factor and immune related biomarker, which can be applied to screen potential effective drugs and target LUAD patients with RA.

In summary, the aforementioned studies yield intriguing findings and contribute fresh insights to the field of big data in biomedical research on cancer. We are appreciative of “Frontiers in Molecular Biosciences” for facilitating this research theme and assembling a diverse group of authors. We also extend our gratitude to the editor for ensuring the quality of the manuscript, the reviewers for their diligent contributions and insightful recommendations, and the journal for providing a vital platform for academic exchange.
